# Research on Miniaturized UHF Sensing Technology for PD Detection in Power Equipment Based on Symmetric Cut Theory

**DOI:** 10.3390/s24113313

**Published:** 2024-05-22

**Authors:** Bowen Xu, Chaoqian Duan, Jiangfan Wang, Lei Zhang, Guozhi Zhang, Guoguang Zhang, Guangke Li

**Affiliations:** 1 Hubei Engineering Research Center for Safety Monitoring of New Energy and Power Grid Equipment, Hubei University of Technology, Wuhan 430068, China; 19932868211@163.com (B.X.); 15171679179@163.com (C.D.); w2862502773@163.com (J.W.); zgg750271126@163.com (G.Z.); 2 Electric Power Research Institute, Guangxi Power Grid Co., Ltd., Nanning 530023, China; zl464873459@163.com; 3 Handan Puxin Electric Power Technology Co., Ltd., Handan 057150, China; lgk3066753001@163.com

**Keywords:** power equipment, partial discharge, ultra-high frequency, miniaturization, symmetric cut theory

## Abstract

In answer to the demand for high sensitivity and miniaturization of ultra-high frequency (UHF) sensors for partial discharge (PD) detection in power equipment, this paper proposes research on miniaturized UHF-sensing technology for PD detection in power equipment based on symmetric cut theory. The symmetric cut theory is applied for the first time to the miniaturization of PD UHF sensors for power equipment. A planar monopole UHF sensor with a size of only 70 mm × 70 mm × 1.6 mm is developed using an exponential asymptotic feed line approach, which is a 50% size reduction. The frequency–response characteristics of the sensor are simulated, optimized and tested; the results show that the standing wave ratio of the sensor developed in this paper is less than 2 in the frequency band from 427 MHz to 1.54 GHz, and less than 5 in the frequency band from 300 MHz to 1.95 GHz; in the 300 MHz~1.5 GHz band; the maximum and average gains of the sensor E-plane are 4.76 dB and 1.02 dB, respectively. Finally, the PD simulation experiment platform for power equipment is built to test the sensor’s sensing performance; the results show that the sensor can effectively detect the PD signals; the sensing sensitivity is improved by about 95% relative to an elliptical monopole UHF sensor.

## 1. Introduction

Partial discharge (PD) is an electrical discharge in which only partial breakdown occurs within the insulation or on the surface of electrical equipment under the action of a locally high electric field [1]. PD characterized by continuous deterioration is one of the main causes of equipment insulation failures. Failure to achieve effective sensing of PD insulation defects may result in serious equipment damage and outages [2,3]. The PD process generates characteristic signals such as high-frequency currents, electromagnetic waves, acoustic waves, optical radiation and thermal radiation [4]. By using sensors to detect these characteristic signals, the online monitoring of the insulation status of electrical equipment can be realized. The ultra-high frequency (UHF) method utilizes UHF sensors to sense the high-frequency electromagnetic wave signals radiated by PD [5], with advantages of high sensitivity and strong anti-interference ability, and is the most widely used PD detection method in the power field [6].

In recent years, scholars at home and abroad have carried out a series of studies in the field of UHF sensors for PD detection. The main common UHF sensors available today are planar monopole sensors, microstrip patch sensors, planar spiral sensors, fractal sensors, etc. [7,8,9,10].

According to the literature, in reference [11], a rectangular planar monopole UHF sensor was designed with dimensions of 200 mm × 187 mm × 1.6 mm for PD detection in substation equipment; this sensor utilized a coplanar waveguide feed mode and enabled PD detection in the 500 MHz to 1120 MHz band; in [12], the authors designed a flexible built-in Archimedes spiral UHF sensor with a diameter of 149 mm, which had good PD detection performance and high sensitivity in the 620 MHz to 3 GHz band; in [13], a Hilbert fractal UHF sensor was designed with dimensions of 124 mm × 122 mm × 0.5 mm, which had three effective detection bands in the 300 MHz to 3 GHz band, covering 73.6% of the PD detection range. However, with the development of intelligent and miniaturized detection technology, these large-size UHF sensors can no longer meet the demand in some application scenarios with limited space and high miniaturization requirements, such as portable or switchgear built-in PD UHF detection scenarios. The large size somewhat hinders the application of UHF sensors in the field of PD detection. Therefore, it is particularly important to conduct miniaturization studies of UHF sensors.

Based on this, this paper proposes research into miniaturized UHF sensing technology for PD detection in power equipment based on symmetric cut theory; the symmetric cut theory is applied to the miniaturization of PD UHF sensors for power equipment, and the planar monopole UHF sensor is developed using an exponential asymptotic feed line approach, the frequency response characteristics of the sensor are simulated, optimized and tested, and the PD simulation experiment platform for the power equipment is built to experimentally verify the sensor’s sensing performance.

## 2. Sensor Design Principles

### 2.1. Planar Monopole Sensor

The planar monopole sensor consists of four parts: dielectric substrate, radiation patch, feed line and ground plate. The planar monopole sensor has the advantages of simple structure, low profile, easy to compound and conform, and with good radiation characteristics [14].

**Figure 1:**
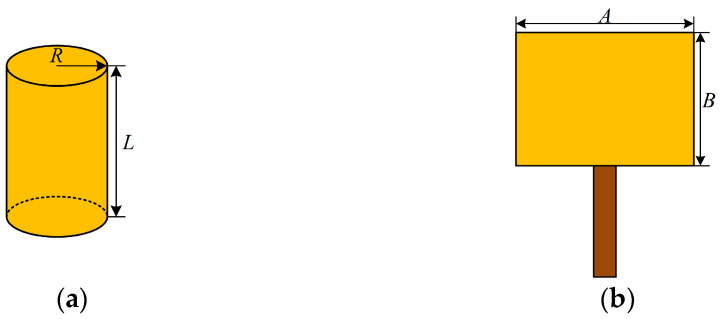
Schematic of the cylindrical oscillator and the main body structure of the rectangular planar monopole sensor: (**a**) cylindrical oscillator; (**b**) rectangular planar monopole sensor’s main body structure.

The basic dimensions of the radiation patch of a planar monopole sensor are determined by the lowest operating frequency it can detect, which can be estimated by the cylinder approximation. When the radiation patch is a rectangle with length *B* and width *A*, the equivalent calculation can be performed by a cylindrical oscillator with a base radius *R* and height *L*. Figure 1 shows the schematic structure of the cylindrical oscillator and the main body of the rectangular planar monopole sensor.

According to the principle of equal area, this gives

(1) 2πRL=BA

The structural dimensions of the cylindrical oscillator and the wavelength corresponding to its lowest operating frequency *λ_L_* satisfy the following equation:

(2) $$L=0.24\lambda_{L}F$$

where *F* is the degree of broadening of the radiation cell, *F* can be expressed as:

(3) $$F=\frac{L}{L+R}$$

Based on Equations (2) and (3), the following conclusions can be drawn.

(4) $$\lambda_{L}=\frac{L}{0.24F}=\frac{L}{0.24\frac{L}{L+R}}=\frac{L+R}{0.24}$$

Then the formula for *f_L_* can be written as

(5) $$f_{L}=\frac{\nu }{\lambda_{L}}=\frac{0.24\nu }{L+R}$$

where *ν* is the propagation speed of electromagnetic waves in different medium.

When the length of the rectangular radiation patch is equal to the height of the cylindrical oscillator, i.e., *B* = *L*, according to Equation (1), there is

(6) $$R=\frac{A}{2\pi }$$

and according to Equation (5), it can be concluded that the lowest operating frequency *f_L_* of the rectangular radiation patch is related to its length *B* and width *A* as

(7) $$f_{L}=\frac{0.48\pi \nu }{2\pi B+A}$$

According to the above theoretical analysis, it can be seen that the larger the size of the radiation patch of the rectangular planar monopole sensor, the better its low-frequency performance, relatively speaking. Expanding the lowest operating frequency of the sensor with limited size is a difficult issue in the miniaturization design of UHF sensors.

### 2.2. Theory of Symmetric Cut Miniaturization

Symmetric cutting is one of the simplest and most direct ways to reduce the size of sensors and is now widely used in the field of miniaturized ultra-wideband (UWB) sensors [15,16]. This method mainly utilizes the magnetic wall effect of the symmetric sensor to reduce the size of the sensor by cutting the symmetric structure sensor in half and adjusting the important parameters [17,18]. The new structure after the symmetric cutting treatment will still retain the resonance characteristics of the initial sensor.

The principle of symmetric cut miniaturization can be explained from the perspective of sensor surface current distribution [19,20]: the surface current of the sensor with a symmetric structure is also symmetrically distributed, and the new sensor formed after symmetric cutting has only half of the structure of the initial sensor, but its surface current distribution is approximately equivalent to the surface current distribution of the initial sensor. As a result, the new sensor formed after symmetrical cutting retains the resonance characteristics of the initial sensor while realizing a halving of its size.

**Figure 2:**
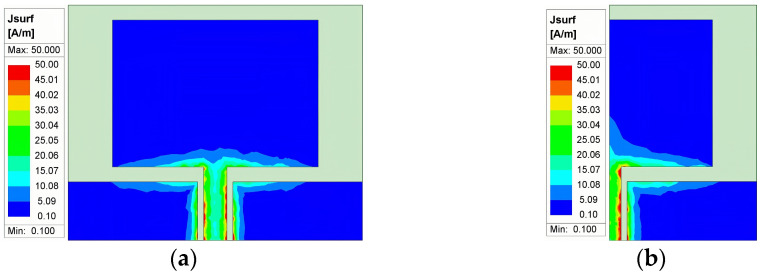
Sensor current distribution before and after symmetric cutting: (**a**) current distribution of the initial sensor; (**b**) current distribution of the sensor after symmetric cutting.

In this paper, a simple rectangular planar monopole sensor model is constructed and symmetrically cut using HFSS electromagnetic wave simulation software. The surface current distribution of the initial planar monopole sensor and the new planar monopole sensor formed after symmetrical cutting at the 1 GHz frequency point is obtained by simulation, as shown in Figure 2.

From Figure 2, it can be seen that the surface current of the initial sensor is symmetrically distributed along the E-plane, and the surface current distribution of the new sensor formed after symmetrical cutting is basically the same as that of the right half plane of the initial sensor. This shows that symmetric cutting does not affect the circuit path of the sensor. In this way, the sensor can be miniaturized without changing its resonance characteristics by means of symmetrical cutting.

However, it should be noted that although the current distribution of the sensor after symmetric cutting is extremely similar to that of the initial sensor, the two are not likely to be identical, and the two current distributions are likely to remain identical only under ideal circumstances.

### 2.3. Coplanar Waveguide Feed

A coplanar waveguide (CPW) feed structure [21] is a microwave coplanar transmission structure. The CPW feed structure consists of a centrally located metal thin-film feed line and metal ground plates on either side of it, with the feed line and the ground plates located in the same plane as the dielectric substrate. Compared to sensors with other feeding modes, sensors fed by the CPW feed structure have the advantages of wide bandwidth, high gain, and omnidirectional radiation.

**Figure 3:**
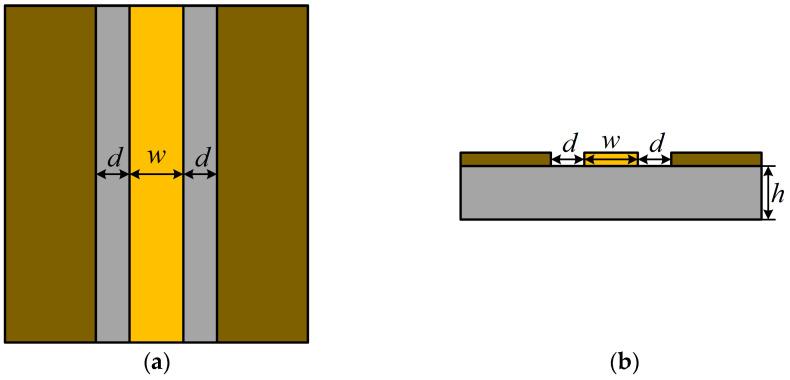
CPW feed structure composition: (**a**) top view of CPW feed structure; (**b**) side view of CPW feed structure.

The CPW feed structure is shown in Figure 3; the width of the feed line is *w*, the spacing between the feed line and the ground plate is *d*, the thickness of the dielectric substrate is *h*.

The characteristic impedance of the CPW feed line can be analyzed by quasi-static analysis with the following formula:

(8) $$Z_{0}=\frac{30\pi K^{\prime }\left(k\right)}{\sqrt{\varepsilon_{re}}K\left(k\right)}$$

where

(9) $$k=d/\left(d+2w\right)$$

(10) $$k^{\prime }=\sqrt{1-k^{2}}$$

(11) $$K^{\prime }\left(k\right)=K\left(k^{\prime }\right)$$

(12) $$\frac{K\left(k\right)}{K^{\prime }\left(k\right)}=\left\{\begin{array}{l} {\left[\frac{1}{\pi }\ln\left(2\frac{1+\sqrt{k^{\prime }}}{1-\sqrt{k^{\prime }}}\right)\right]}^{-1}\;\;\left(0\leq k<0.7\right)\\ \frac{1}{\pi }\ln\left(2\frac{1+\sqrt{k}}{1-\sqrt{k}}\right)\;\;\;\;\;\;\left(0.7\leq k\leq 1\right) \end{array}\right.$$

where *K*(*k*) is the first type of fully elliptic function, *K*′(*k*) is the first type of fully elliptic residual function, and *ε_re_* is the equivalent dielectric constant of the dielectric substrate.

Based on the above reasoning, it can be seen that the characteristic impedance of a coplanar waveguide feed line can be calculated when the CPW feed line width *w*, the spacing between the CPW feed line and the ground plate *d*, the height of the dielectric substrate *h*, and the equivalent dielectric constant *ε_re_* of the dielectric substrate are known.

## 3. UHF Sensor Design and Optimization

### 3.1. Sensor Design

#### 3.1.1. Sensor Body Design

The design of the initial dimensions of the sensor is based on the lowest operating frequency of the operating band. Given that the primary frequency bands in which PD radiates a high-frequency electromagnetic wave energy span from 300 MHz to 1.5 GHz [22], in this paper, 300 MHz is chosen as the lowest operating frequency of the designed planar monopole sensor.

**Figure 4:**
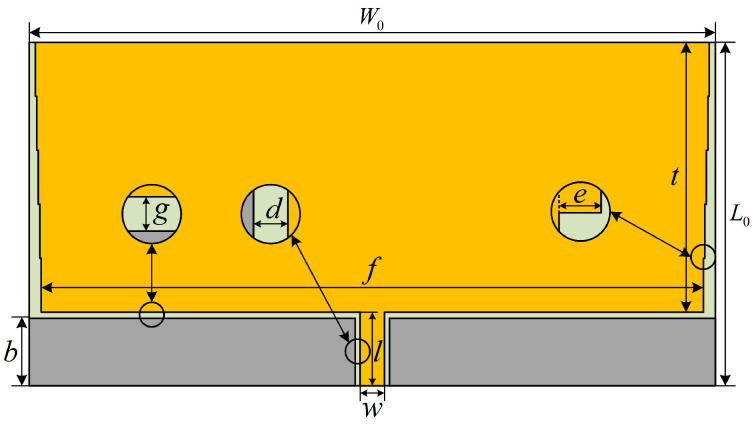
Initial structure of the planar monopole sensor.

Figure 4 shows the initial structural model of the planar monopole sensor designed in this paper, which consists of a dielectric substrate, a rectangular radiation patch, a CPW feed line and a metal ground plate. The rectangular radiation patch introduces an inverted stepped gradient structure [23] to realize multistage matching and broaden the operating frequency band of the sensor. In addition, the stepped gradient structure can also extend the current path and improve the low-frequency performance of the sensor.

The various parameters of the initial structure of the planar monopole UHF sensor designed in this paper can be obtained from the formula of the planar monopole sensor and the formula of the CPW feed line characteristic impedance, as shown in Table 1. The sensor dielectric substrate is made of FR4, the most commonly used material, with a relative dielectric constant of 4.4; the thickness of the dielectric substrate *h* is taken as 1.6 mm.

The sensor has a longitudinal length of 70 mm and a transverse width of 140 mm, which are large dimensions and need to be further optimized.

#### 3.1.2. Symmetric Cut Miniaturization Optimization

**Figure 5:**
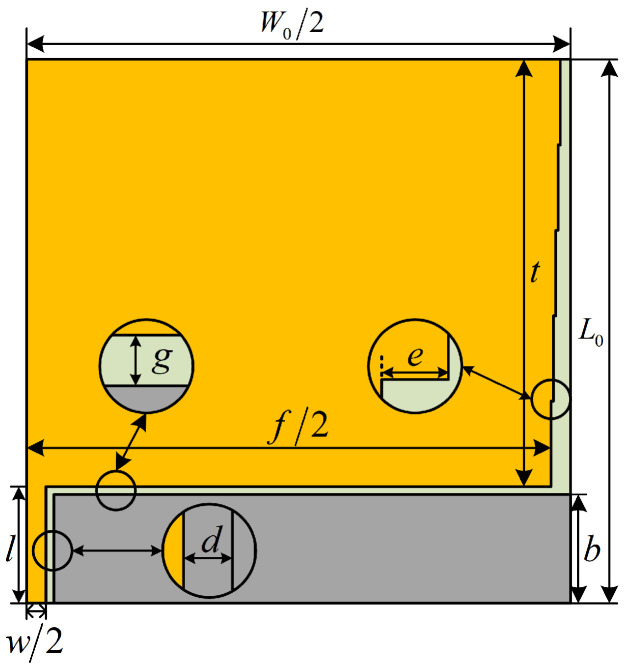
Sensor structure after symmetric cutting process.

According to Figure 4, it can be seen that the planar monopole UHF sensor designed in this paper is a planar symmetric structure, so it is cut symmetrically along the E-plane and the right half plane is retained, as shown in Figure 5. After the symmetric cutting process, the transverse width of the sensor is changed from *W*_0_ to *W*_0_/2, and the width of the CPW feed line is changed from *w* to *w*/2, and the overall size of the sensor is reduced from 70 mm × 140 mm to 70 mm × 70 mm, which is a 50% reduction in size, realizing the miniaturization of the sensor.

#### 3.1.3. Asymptotic Treatment of CPW Feed Line

In order to reduce the energy reflection at the input of the UHF sensor and improve the signal transmission efficiency, the CPW feed line is optimized by asymptotic processing in this paper.

**Figure 6:**
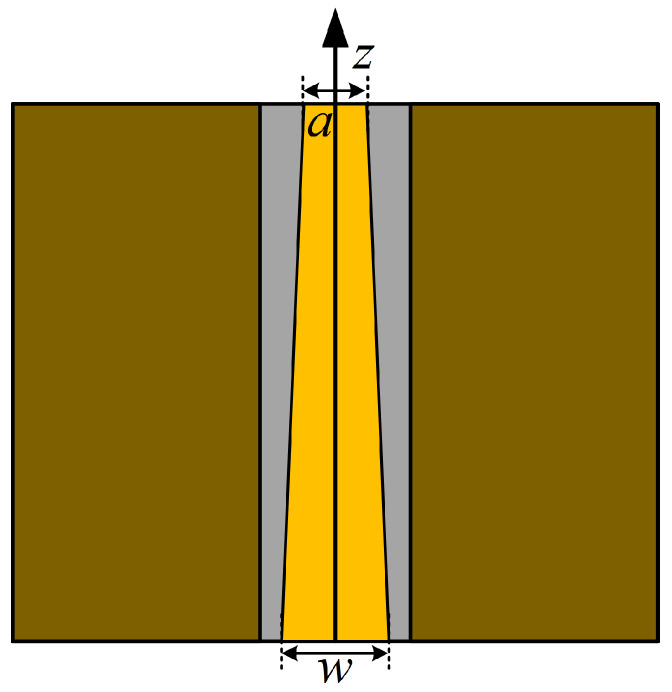
Schematic diagram of the exponential asymptotic CPW feed line structure.

Commonly used forms of asymptotes are exponential asymptotes, hyperbolic asymptotes, Bessel curves, and parabolas. The exponential asymptotes provided the best impedance matching [24]. Figure 6 shows the schematic structure of the exponential asymptotic CPW feed line.

The characteristic impedance *Z_c_* of the CPW feed line varies with an exponential law in the direction of the length of the feed line, and is given by:

(13) $$Z_{c}\left(z\right)=Z_{0}e^{\alpha z}\left(0<z<l\right)$$

where *α* is the characteristic impedance transformation constant, and

(14) $$Z_{c}\left(0\right)=Z_{0}$$

(15) $$Z_{c}\left(l\right)=Z_{0}e^{\alpha l}=Z_{L}$$

where *Z*_0_ is the characteristic impedance at the input port of the CPW feed line and *Z_L_* is the input impedance of the load connected at the termination of the CPW feed line and, according to Equations (14) and (15), it can be concluded that:

(16) $$\alpha =\frac{1}{l}\ln\left(\frac{Z_{L}}{Z_{0}}\right)$$

Combined with the theory of small reflections, the formula for the reflection coefficient Γ at the input of the CPW feed line can be derived:

(17) $$\Gamma =\frac{\lambda }{8\pi l}\ln\frac{Z_{L}}{Z_{0}}$$

Theoretically, the longer the length *l* of the CPW feed line, the smaller the reflection coefficient at the incident end, and the better the impedance matching effect. After selecting the length *l* of the feed line, the input reflection coefficient and the impedance transformation constant can be calculated by the above inference. However, too long a feed line will lead to the overall size of the sensor increasing, so after comprehensive consideration of the size of the sensor and the performance of the mutual constraints, and finally *l* = 15 mm. In addition, the exponential asymptotic treatment of the CPW feed line derives a new parameter, i.e., the width *a* of the top end of the CPW feed line.

### 3.2. Optimization of Sensor Parameters

It has been shown that adopting incomplete coverage of the ground plane can broaden the operating band of the sensor [25,26]; based on this, a new parameter is introduced in this paper: the distance *c* between the ground plate and the outer edge of the dielectric substrate. In addition, the parameter *a* is closely related to the impedance matching of the sensor, and the incremental width *e* of each step of the inverted stepped gradient structure has an effect on the low-frequency performance of the sensor. Therefore, in this paper, a parameter-by-parameter optimization approach is adopted to optimize the above three parameters.

**Figure 7:**
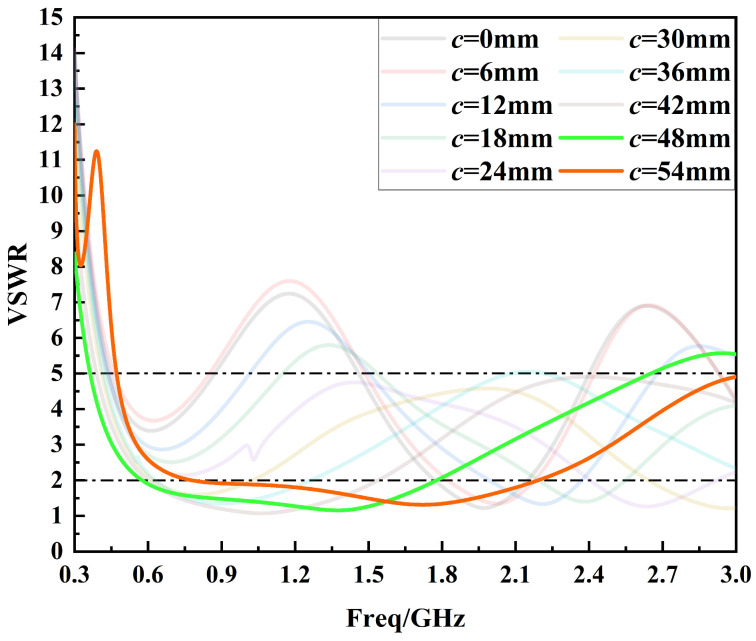
Voltage standing wave ratio curves for the first optimization of parameter *c*.

First, the parameter *c* is optimized for the first time in the range of 0 to 54 mm in steps of 6 mm, as shown in Figure 7. By analyzing Figure 7, it can be seen that the sensor performance gradually becomes better in the 0.3 GHz to 1.5 GHz band as the parameter *c* is varied from 0 to 48 mm. The sensor performance is optimized when *c* = 48 mm and deteriorates instead when *c* = 54 mm. Therefore, the optimal interval for parameter *c* is 48 mm to 54 mm.

**Figure 8:**
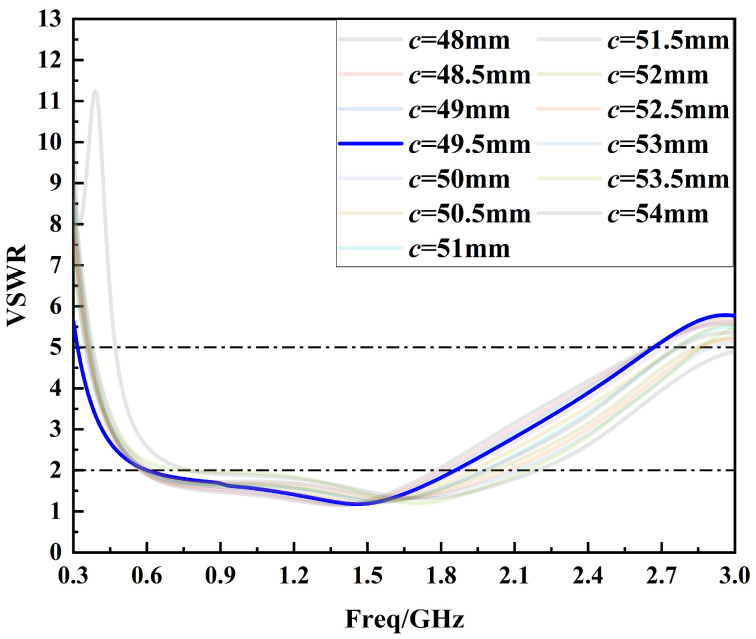
Voltage standing wave ratio curves for the second optimization of parameter *c*.

After determining the optimal interval of parameter *c*, the second optimization of parameter *c* is carried out within the optimal interval of 48 mm to 54 mm in steps of 0.5 mm, as shown in Figure 8. By analyzing Figure 8, it can be seen that when the parameter *c* = 49.5 mm, the sensor voltage standing wave ratio curve is less than 6 in the frequency band from 0.3 GHz to 1.5 GHz and less than 2 in most of the frequency bands, and the performance of the sensor reaches the optimum. Therefore, the optimum value of parameter *c* is 49.5 mm.

**Figure 9:**
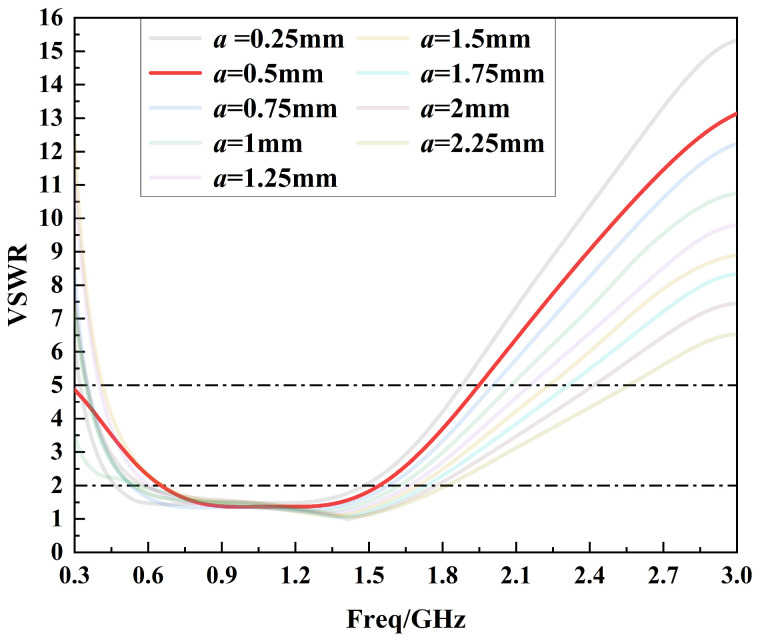
Voltage standing wave ratio curves for the optimization of parameter *a*.

After determining the optimal value of parameter *c*, parameter *a* is optimized in the range of 0.25 mm to 2.5 mm in steps of 0.25 mm, as shown in Figure 9. From Figure 9, it can be seen that the low-frequency performance of the sensor fluctuates greatly while the high-frequency performance tends to deteriorate when the parameter *a* is gradually changed from 2.25 mm to 0.25 mm. In order to balance the low-frequency performance and high-frequency performance of the sensor, the optimal value of the parameter *a* is finally chosen to be 0.5 mm. And, at this point, the sensor voltage standing wave ratio curve is less than 5 in the 0.3 GHz to 1.5 GHz band.

**Figure 10:**
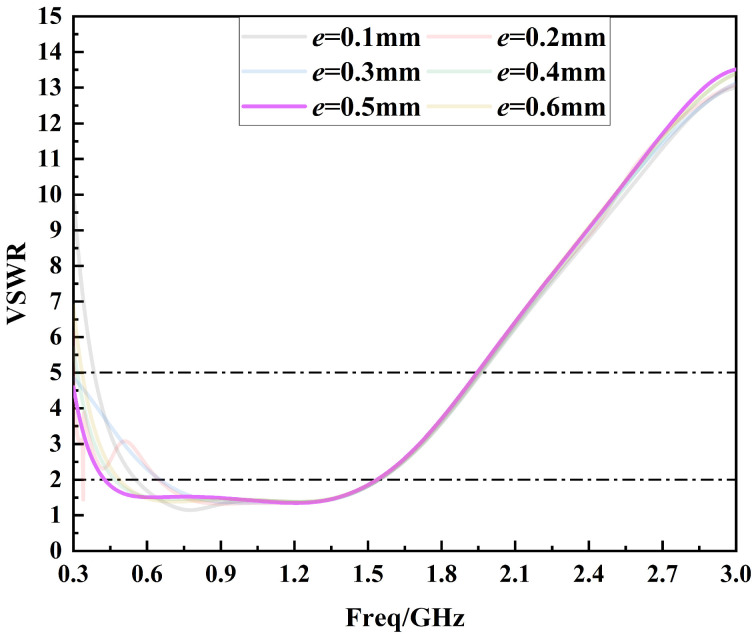
Voltage standing wave ratio curves for the optimization of parameter *e*.

After determining the optimal value of parameter *a*, the step width *e* is optimized in the range of 0.1 mm to 0.6 mm in steps of 0.1 mm, as shown in Figure 10. From Figure 10, it can be seen that the parameter *e* mainly affects the low-frequency performance of the sensor, while it has less influence on the high-frequency performance. And the sensor performance is optimized when *e* = 0.5 mm. At this time, the voltage standing wave ratio curve of the sensor is less than 5 in the frequency band from 0.3 GHz to 1.5 GHz, and the low-frequency performance of the sensor is also best.

Through the above simulation optimization, the optimal values of the three parameters *c*, *a* and *e* of the sensor are finally determined to be 49.5 mm, 0.5 mm and 0.5 mm respectively.

**Figure 11:**
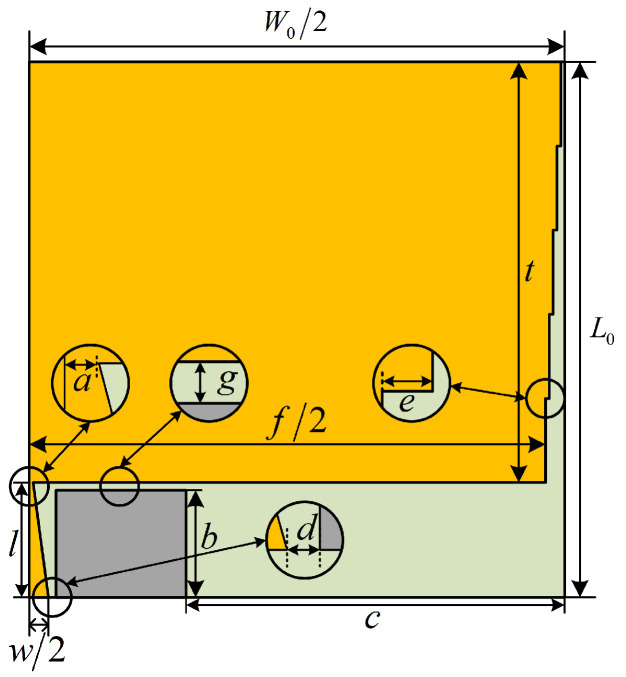
Final model of the sensor.

The final model of the sensor is shown in Figure 11 and the final parameter information of the sensor is shown in Table 2.

**Figure 12:**
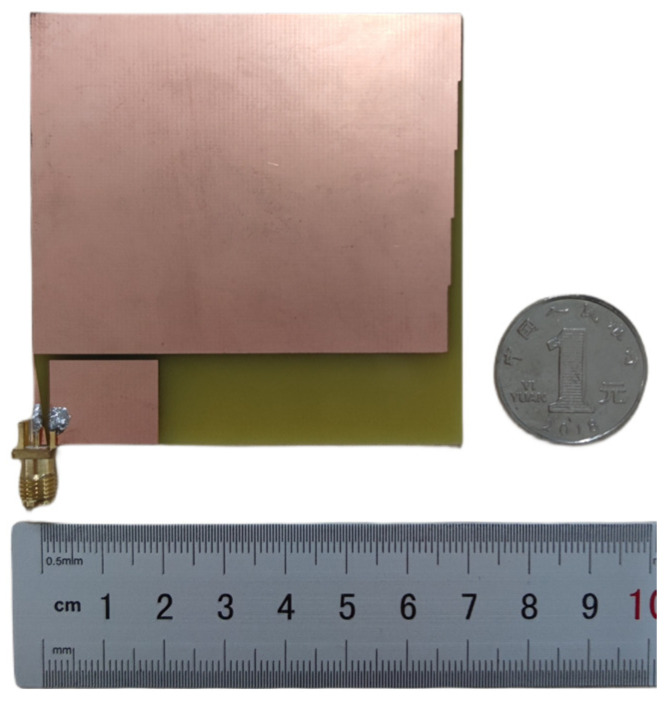
Photograph of the sensor designed in this paper.

Pilot fabrication of the optimized sensor model is carried out, as shown in Figure 12. The sensor body part and the feed structure part are made of a metallic copper material of 18 um thickness, and the metallic copper layer is plated onto the FR4 dielectric substrate with a thickness of 1.6 mm using a printing process. And the sensor feed structure part is soldered to a 50-ohm SMA connector.

## 4. Sensor Performance Analysis

### 4.1. Voltage Standing Wave Ratio

The degree of matching between the characteristic impedance of the transmission line and the input impedance of the sensor is usually characterized by the voltage standing wave ratio (VSWR), which is the ratio of the voltage of the wave belly to that of the wave node, and is expressed by the formula:

(18) $$VSWR=\frac{{\left|U\right|}_{\max}}{{\left|U\right|}_{\min}}=\frac{1+\left|\Gamma \right|}{1-\left|\Gamma \right|}$$

where Γ is the reflection coefficient at the sensor input, indicating the ratio of the reflected wave voltage to the incident wave voltage.

When VSWR = 1, it indicates that the input impedance of the sensor and the characteristic impedance of the feed line are perfectly matched and the signal transmission is an ideal reflection-free state; however, when VSWR is infinite, it indicates that the input impedance of the sensor and the characteristic impedance of the feed line are completely mismatched, and the incident signals at the input port is completely reflected.

It is generally believed that when the sensor VSWR curve is less than 2, the sensor receives electromagnetic signals with good performance. However, in fact, the UHF sensor is closer to the PD source, which can effectively receive electromagnetic radiation, so it is generally believed that the sensor VSWR curve is less than or equal to 5 in the PD detection frequency band to meet the requirements.

**Figure 13:**
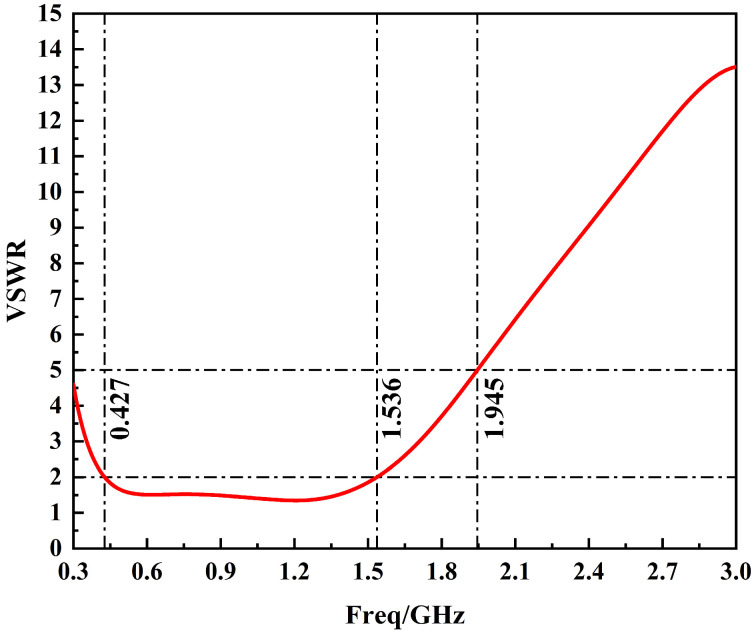
Simulated VSWR curve of the sensor designed in this paper.

The VSWR curve of the sensor is simulated using HFSS in the 300 MHz~3 GHz band as shown in Figure 13. The results show that the sensor VSWR curve is less than 5 in the 300 MHz~1.95 GHz band, and less than 2 in the 427 MHz~1.54 GHz band. The simulation results of the voltage standing wave ratio curve of the sensor show that the impedance of the sensor is well matched.

**Figure 14:**
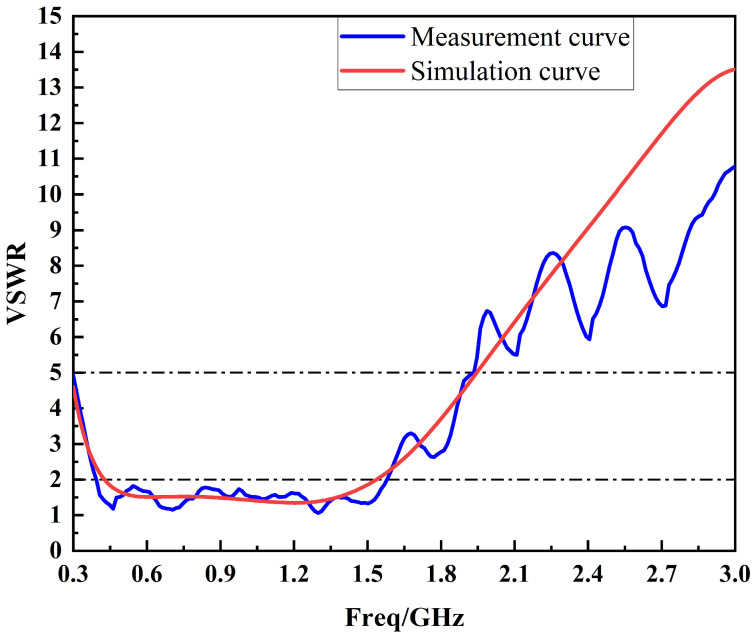
Comparison of VSWR simulation curve and measured curve.

After the simulation and analysis, the VSWR curve of the sensor is tested practically in the frequency range of 300 MHz~3 GHz using the vector network analyzer from Agilent, Santa Clara, CA, USA (E5063A), as shown in Figure 14.

It can be seen from Figure 14 that the overall trend of the measured VSWR curve and the simulated VSWR curve is consistent. In the low-frequency band, the simulated VSWR curve is smoother, while the measured VSWR curve shows slight fluctuations. And in the high-frequency band, the simulated VSWR curve shows a rapid rising trend, while the measured VSWR curve shows an oscillating rising trend. The differences between measured and simulated performances are mainly related to the welding process, the sensor’s working environment and other factors. Overall, the measured and simulated performances of the sensor are similar, and the measured performance is better.

### 4.2. Gain

The gain of a sensor in a given direction is the ratio of the actual radiation intensity of the sensor in that direction to that of an ideal point source when the system transmit power is certain [27]. The gain of the sensor is used to quantitatively characterize the extent to which the sensor concentrates the input power to radiate. The gain *G* of the sensor is closely related to its directivity coefficient *D* and radiation efficiency *e_r_*:

(19) $$G=De_{r}$$

where *e_r_* can be calculated by the following equation:

(20) $$e_{r}=\frac{P_{r}}{P_{in}}=\frac{P_{r}}{P_{r}+P_{\sigma }}$$

where *P_r_* is the radiated power of the sensor, *P_in_* is the incident power of the sensor, and *P_σ_* is the ohmic loss of power.

The gain of a sensor is generally measured in dB:

(21) $$G_{dB}=10\mathrm{lg}\left(G\right)$$

**Figure 15:**
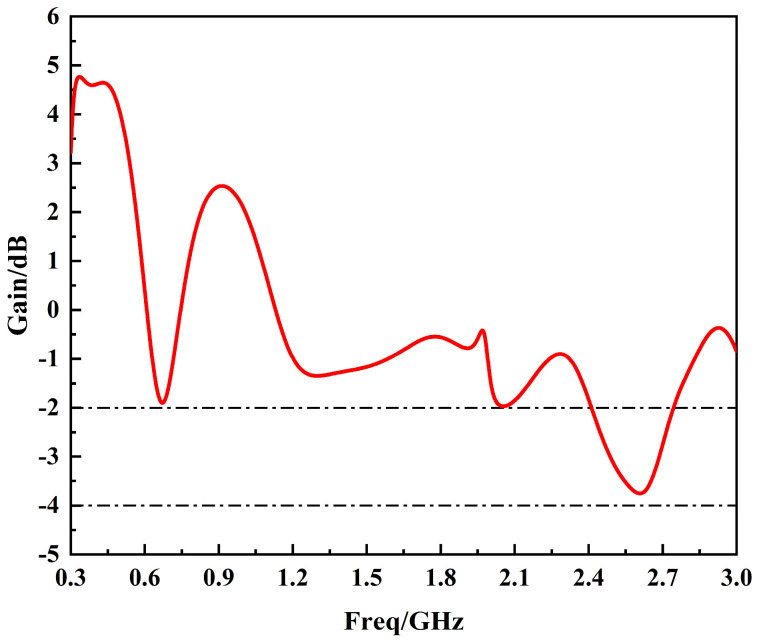
Maximum gain curve of the E-plane of the sensor designed in this paper.

When analyzing the gain of the sensor, the focus should be on the maximum gain of the sensor, i.e., the gain of the sensor in the direction of maximum radiation. In this paper, the maximum gain curve of the E-plane of the sensor is analyzed by simulation, as shown in Figure 15.

By analyzing Figure 15, it becomes evident that the maximum gain curve of the E-plane of the sensor is above −2 dB in the 300 MHz~1.5 GHz band, and is above −4 dB in the 300 MHz~3 GHz band, with the maximum gain curve appearing in the −2° direction. In addition, the maximum gain in the E-plane of the sensor is 4.76 dB in both the 300 MHz~1.5 GHz band and the 300 MHz~3 GHz band. Moreover, in the frequency band of 300 MHz~1.5 GHz, the average gain of the E-plane of the sensor is 1.02 dB; and in the frequency band of 300 MHz~3 GHz, the average gain of the E-plane of the sensor is −0.37 dB. In general, the gain performance of the sensor designed in this paper is excellent, which is in line with the basic requirements of a UHF sensor for PD detection in power equipment.

### 4.3. PD Perception Experimental Test

**Figure 16:**
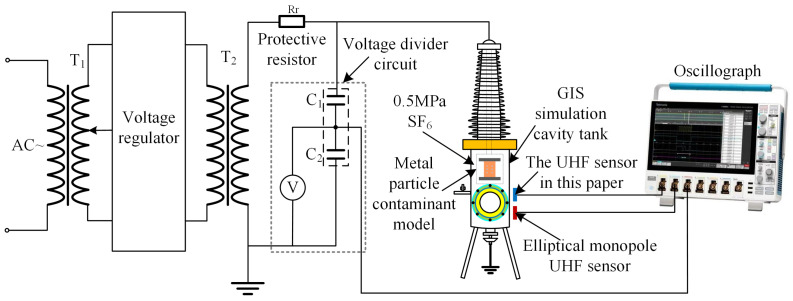
Circuit diagram of PD detection simulation experiment platform.

**Figure 17:**
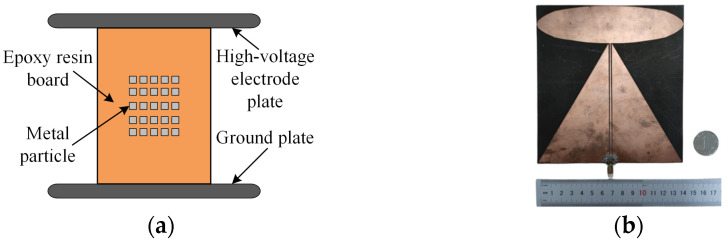
Schematic of metal particle contaminant model and comparison sensor: (**a**) metal particle contaminant model; (**b**) elliptical monopole sensor for comparison.

In order to test the actual performance of the designed planar monopole UHF sensor in detecting PD signals, a GIS PD simulation experimental platform is built in this paper, as shown in Figure 16. In this case, the UHF sensors to be tested are placed outside the Plexiglas window, the PD defect is modeled as a metallic particulate fouling defect commonly found in GIS, as shown in Figure 17a. The comparison sensor is a high-performance elliptical monopole UHF sensor, as described in the literature [28], and shown in Figure 17b. The comparison sensor has the same operating frequency band and similar radiation characteristics as the sensor designed in this paper. The VSWR curve of the comparison sensor remains below 3 in the frequency band from 300 MHz to 3 GHz and below 2 in the frequency band from 650 MHz to 3 GHz. And the H-plane and E-plane directional patterns of the comparison sensor have an inverted “8” shape at a number of frequency points.

**Figure 18:**
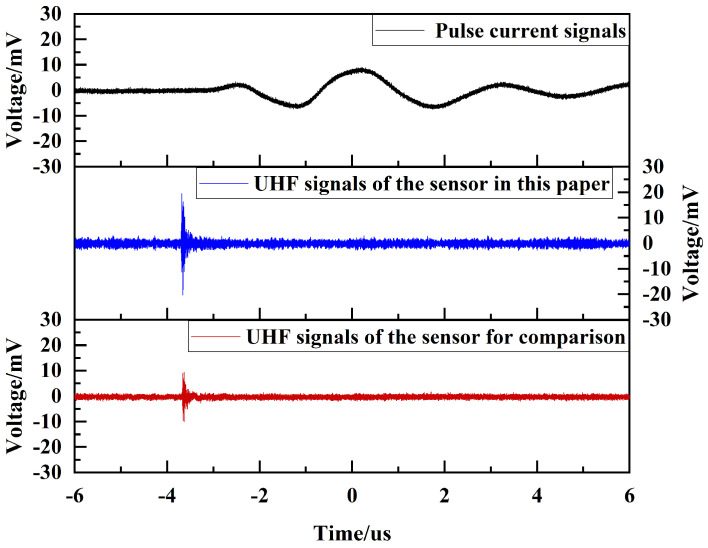
PD UHF time-domain signals detected by sensors.

The PD platform is pressurized using the step-up method, and the starting discharge voltage of the PD defect is measured to be 15 kV and the flashover voltage to be 54 kV. The PD UHF signals detected by the UHF sensor designed in this paper and the comparison UHF sensor at a test voltage of 16 kV and a discharge of 6.4 pC are shown in Figure 18. As can be seen from Figure 18, the detection sensitivity of the UHF sensor designed in this paper is significantly better than that of the comparison UHF sensor.

**Figure 19:**
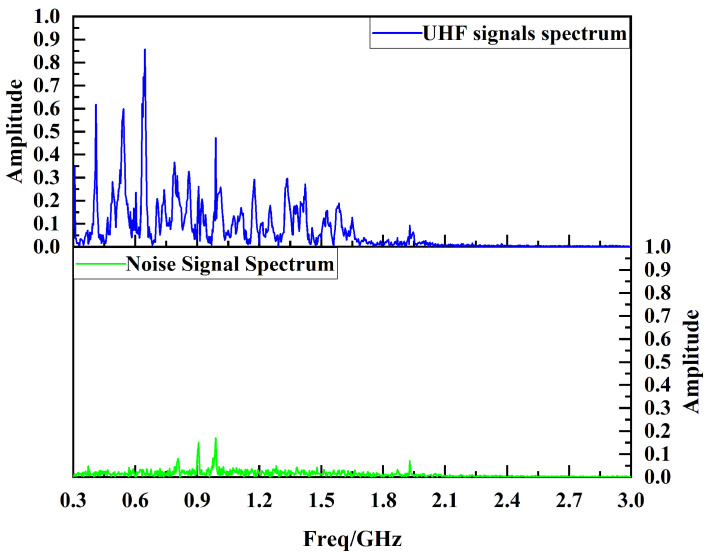
Spectrum of UHF signals and noise signals.

The PD UHF signals and the background noise signals detected by the UHF sensor designed in this paper are analyzed by Fourier spectrum, and the results are shown in Figure 19. The background noise signals mainly interfere with the communication band near 900 MHz. The UHF signal energy detected by the UHF sensor designed in this paper is mainly concentrated in the frequency band of 390 MHz~1.65 GHz, which is basically consistent with the effective frequency band of the UHF sensor designed in this paper. And the band energy is significantly larger than the noise energy. Spectral analysis illustrates that the sensor designed in this paper has excellent PD radiation UHF signal sensing capability.

**Figure 20:**
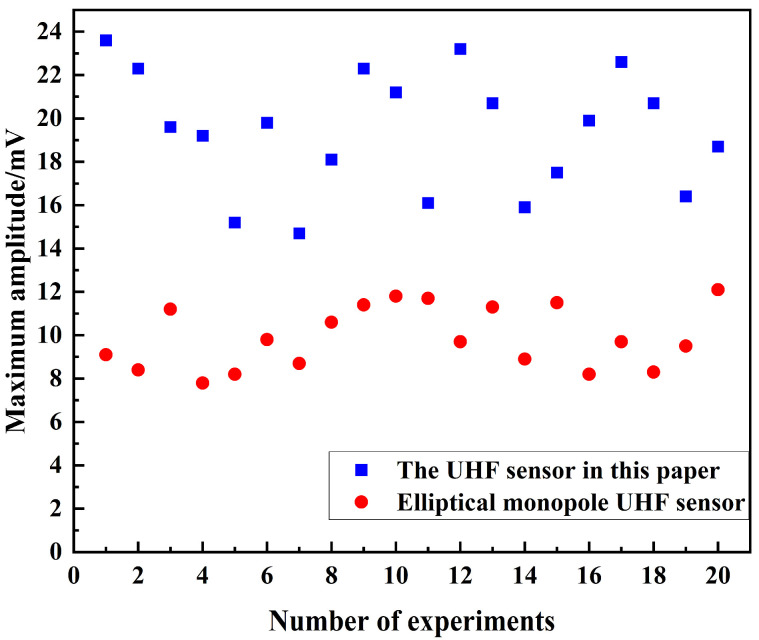
Statistical data from multiple PD experiments.

Further, in order to more accurately verify the sensing performance of the UHF sensor designed in this paper, 20 PD experiments were randomly carried out in the test voltage range of 15.6 kV to 19.6 kV, and the experimental results are shown in Figure 20. As can be seen from Figure 20, in the 20 PD experiments, both the UHF sensor designed in this paper and the comparison UHF sensor were able to detect PD UHF signals with signal amplitudes in the range of 7 mV~24 mV. However, the amplitude of the PD UHF signals detected by the UHF sensor designed in this paper is significantly larger than that of the comparison UHF sensor.

The results of the 20 PD experiments were analyzed, and the average amplitude of the PD UHF signals was calculated by taking the data of 4 adjacent experiments as a group, and the percentage improvement in the average amplitude of the PD UHF signals detected by the UHF sensor designed in this paper compared to the comparison sensor was also calculated. The data are shown in Table 3.

As can be seen from Table 3, the average amplitude of the PD UHF signals detected by the UHF sensor designed in this paper is higher than that of the comparison UHF sensor in all the five data comparisons, with the maximum percentage increase in detection amplitude reaching 131.98% and the minimum being 80%. Moreover, the average amplitude of the PD UHF signals detected by the UHF sensor designed in this paper in 20 PD experiments is 95.91% higher than that of the comparison UHF sensor. The comparison of experimental data illustrates that the performance of the UHF sensor designed in this paper is significantly better than the comparison UHF sensor.

## 5. Conclusions

In order to meet the demand for high sensitivity and miniaturization of UHF sensors for GIS PD detection, this paper proposes research on miniaturized UHF sensing technology for PD in power equipment based on symmetric cut theory, which is the first time that the symmetric cut theory has been used for the miniaturization of UHF sensors. The optimization of a sensor using an exponential asymptotic feed line approach resulted in the design of a millimeter-scale UHF sensor with excellent performance. The conclusions are as follows:

- By miniaturizing the sensor through the symmetric cut miniaturization theory, the size of the sensor is reduced by 50% to only 70 mm × 70 mm with a thickness of 1.6 mm. The sensor has a wide detection band with a VSWR of less than 2 in the 427 MHz~1.54 GHz band and a VSWR of less than 5 in the 300 MHz~1.95 GHz band, which can cover the main frequency band of PD detection; in the 300 MHz~1.5 GHz band, the maximum gain and average gain of the E-plane of the sensor are 4.76 dB and 1.02 dB, respectively, and the performance of the sensor gain is good.
- The experimental results show that the UHF sensor designed by the symmetric cut theory in this paper has excellent PD sensing performance on the basis of a substantial reduction of the sensor structure size; compared to the highly sensitive elliptical monopole UHF sensor, the sensing sensitivity of the UHF sensor designed in this paper is improved by about 95%.

## Figures and Tables

**Table 1:** Geometric parameters of the initial sensor.

Parameter Name	Notation	Value (mm)
Length of patch	*t*	55
Width of patch	*f*	135
Width of steps	*e*	0.3
Height of ground plate	*b*	14
Spacing between feed line and ground plate	*d*	0.5
Gap between patch and ground plate	*g*	1
Length of feed line	*l*	15
Width of feed line	*w*	5

**Table 2:** Final parameter information of the sensor.

Parameter Name	Notation	Value (mm)
Length of patch	*t*	55
Width of patch	*f*/2	67.5
Width of steps	*e*	0.5
Height of ground plate	*b*	14
Spacing between feed line and ground plate	*d*	0.5
Gap between patch and ground plate	*g*	1
Length of feed line	*l*	15
Bottom width of the feed line	*w*/2	2.5
Top width of the feed line	*a*	0.5
Distance between ground plate and outer edge of the dielectric substrate	*c*	49.5

**Table 3:** PD UHF signals’ average amplitude comparison.

Number of Experiments	Average Amplitude of the Sensor in This Paper (mV)	Average Amplitude of the Comparison Sensor (mV)	Percentage Improvement
1~4	21.18	9.13	131.98%
5~8	16.95	9.33	81.67%
9~12	20.07	11.15	80%
13~16	18.5	9.98	85.37%
17~20	20.14	9.9	103.47%
1~20	19.385	9.895	95.91%

## Data Availability

The data presented in the article is original and has not been inappropriately selected, manipulated, enhanced, or fabricated by us.
